# AmrZ is a major determinant of c-di-GMP levels in *Pseudomonas fluorescens* F113

**DOI:** 10.1038/s41598-018-20419-9

**Published:** 2018-01-31

**Authors:** Candela Muriel, Eva Arrebola, Miguel Redondo-Nieto, Francisco Martínez-Granero, Blanca Jalvo, Sebastian Pfeilmeier, Esther Blanco-Romero, Irene Baena, Jacob G. Malone, Rafael Rivilla, Marta Martín

**Affiliations:** 1 Departamento de Biología, Universidad Autónoma de Madrid, Darwin, 2, 28034 Madrid, Spain; 2 Department of Molecular Microbiology, John Innes Centre, Colney Lane, NR47UH Norwich, UK

## Abstract

The transcriptional regulator AmrZ is a global regulatory protein conserved within the pseudomonads. AmrZ can act both as a positive and a negative regulator of gene expression, controlling many genes implicated in environmental adaption. Regulated traits include motility, iron homeostasis, exopolysaccharides production and the ability to form biofilms. In *Pseudomonas fluorescens* F113, an *amrZ* mutant presents a pleiotropic phenotype, showing increased swimming motility, decreased biofilm formation and very limited ability for competitive colonization of rhizosphere, its natural habitat. It also shows different colony morphology and binding of the dye Congo Red. The *amrZ* mutant presents severely reduced levels of the messenger molecule cyclic-di-GMP (c-di-GMP), which is consistent with the motility and biofilm formation phenotypes. Most of the genes encoding proteins with diguanylate cyclase (DGCs) or phosphodiesterase (PDEs) domains, implicated in c-di-GMP turnover in this bacterium, appear to be regulated by AmrZ. Phenotypic analysis of eight mutants in genes shown to be directly regulated by AmrZ and encoding c-di-GMP related enzymes, showed that seven of them were altered in motility and/or biofilm formation. The results presented here show that in *P. fluorescens*, AmrZ determines c-di-GMP levels through the regulation of a complex network of genes encoding DGCs and PDEs.

## Introduction

AmrZ is a highly conserved transcriptional regulator within the pseudomonads^1^. It was originally identified as a transcriptional activator of alginate production in *Pseudomonas aeruginosa* and named AlgZ^2^. It was later shown to regulate also twitching motility and the name was changed to AmrZ (alginate and motility regulator)^3^. Its implication in regulation of motility was extended by the finding of AmrZ as a repressor of flagellar synthesis in cystic fibrosis mucoid isolates of *P. aeruginosa*. This repression is exerted through transcriptional repression of the master regulator of flagella synthesis *fleQ*^4^. Regulation of flagella synthesis by AmrZ through repression of *fleQ* was also observed in the plant-associated soil microbe *P. fluorescens* F113^5^. AmrZ acts therefore as an activator and a repressor of the transcription of different genes. ChIP-Seq analysis both in *P. aeruginosa*^6^ and *P. fluorescens*^1^ has shown that AmrZ binds to hundreds of regions in both genomes and has been considered therefore to be a global regulator. Many of the AmrZ regulated genes are implicated in environmental adaption, and besides motility and alginate production, AmrZ regulates, among others, genes implicated in iron homeostasis, virulence, signal transduction and c-di-GMP production both in *P. aeruginosa* and *P. fluorescens*^1,5,7^. AmrZ has also been shown to transcriptionally repress cellulose production, which is c-di-GMP stimulated, in *P. syringae*^8^.

c-di-GMP is a messenger molecule implicated in bacterial adaption and defines bacterial life-styles. In this sense, the cytoplasmic levels of c-di-GMP are involved in the transition from sessile to planktonic life-style and, in the case of pathogens from acute to chronic infection^9^. c-di-GMP levels are maintained by the opposing activity of diguanylate cyclases and phosphodiesterases^10^. The former are proteins with GGDEF domains while the later are proteins containing EAL or HD-GYP domains^11–13^. GGDEF and EAL domains are often present in the same protein^14^. In these cases, proteins can either be bi-functional^15^ or only one of the domains possesses catalytic activity^16^. Other proteins implicated in c-di-GMP related functions contain sensor or receptor domains, such as the PilZ domain^17^, among others. We have previously shown by ChIP-Seq that in *P. fluorescens* F113, fourteen genes encoding proteins that contain GGDEF, EAL or HD-GYP domains are predicted to be targets of AmrZ^1^.

The aim of this work was to investigate the role of AmrZ in the regulation of the cytoplasmic levels of c-di-GMP in *P. fluorescens* F113 and the impact on adaptive traits such as motility and biofilm formation. We have used a combination of RNA-Seq and the phenotypic analysis of mutants affected in AmrZ and in c-di-GMP related genes, confirmed to be regulated by this global transcriptional regulator.

## Results

### AmrZ affects motility, biofilm formation and competitive rhizosphere colonization

**Figure 1:**
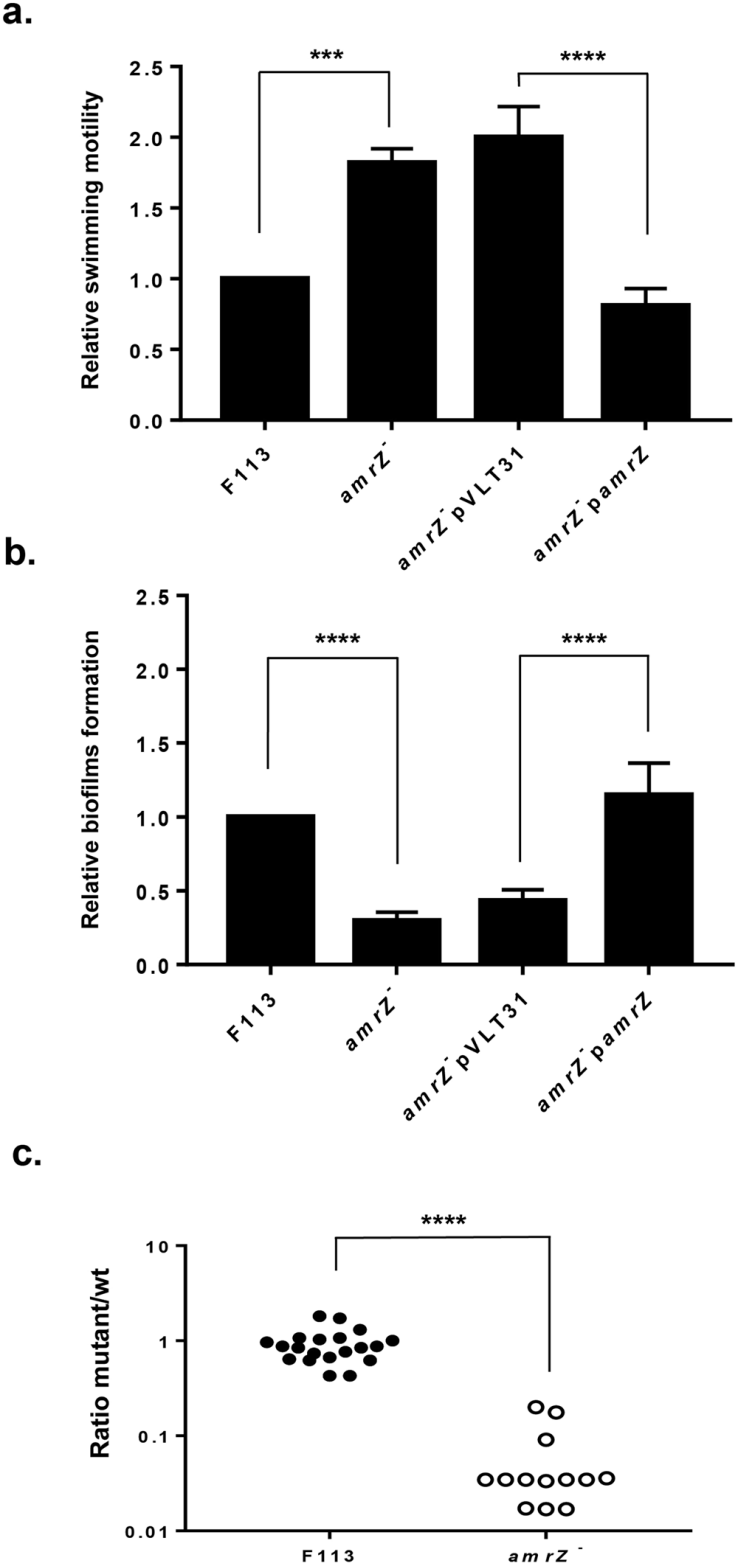
AmrZ affects motility, biofilm formation and competitive rhizosphere colonization. (**a**) Swimming motility phenotypes of *P. fluorescens* F113, its *amrZ* mutant and complementation analysis of the mutant. 24 h haloes on SA with 0.3 agar were measured and referred to the control strains. Averages and standard deviation of three biological replicas with three technical replications are presented. (**b**) Biofilm formation (attachment) phenotypes of *P. fluorescens* F113, its *amrZ* mutant and complementation analysis of the mutant. Crystal violet staining the biomass attached to the walls of microtiter plates after 2 h of incubation was determined at OD_590_ and referred to the control strains. Averages and standard deviations of three biological replicates with sixteen technical replications are presented. (**c**) Competitive rhizosphere colonization of *P. fluorescens* F113 and its *amrZ* mutant. Bacterial colonies recovered from the rhizosphere after one-week inoculation were tested for antibiotic resistance to distinguish the control and the tester strains and counted. Percentages of tester colonies referred to total colonies are represented in a logarithmic scale. Each experiment was repeated three times, with eight plants per experiment. Each dot represents results from a single plant. Asterisks represent statistical significance of the data: ***p < 0.001; ****p < 0.0001.

We have previously shown that AmrZ affects motility since an *amrZ* mutant is hypermotile due to enhanced production of flagellar components^1,4^. As shown in Fig. 1a, ectopic expression of a wild-type (wt) copy of the *amrZ* gene restores wild-type motility to the mutant strain. When grown in King’s B liquid medium under shaking conditions, the *amrZ* mutant does not form a pellicle on the tube surface compared to the pellicle produced by the wild-type strain F113 (Supplementary Fig. 1) suggesting a defect in biofilm formation. For this reason, we performed an assay to determine attachment to plastic microtiter plates. As shown in Fig. 1b, attachment to plastic in microtiter plates is severely reduced in the *amrZ* mutant when compared with the wt. Complementation of the mutant with a plasmid expressing *amrZ* from a constitutive promoter restored attachment to the wild-type levels. Taken together, these results show that AmrZ is required for formation of wild-type biofilm levels.

We have previously shown that hypermotile F113 mutants have increased ability for competitive rhizosphere colonization, even when they are defective for biofilm formation^18^. However, when rhizosphere colonization analyses were performed to compare the *amrZ* mutant with the wild-type strain (Fig. 1c), the *amrZ* mutant was severely impaired in rhizosphere competitive colonization, indicating that the function of AmrZ is required for fitness in the rhizosphere environment.

### AmrZ regulates exopolysaccharide production and c-di-GMP levels

**Figure 2:**
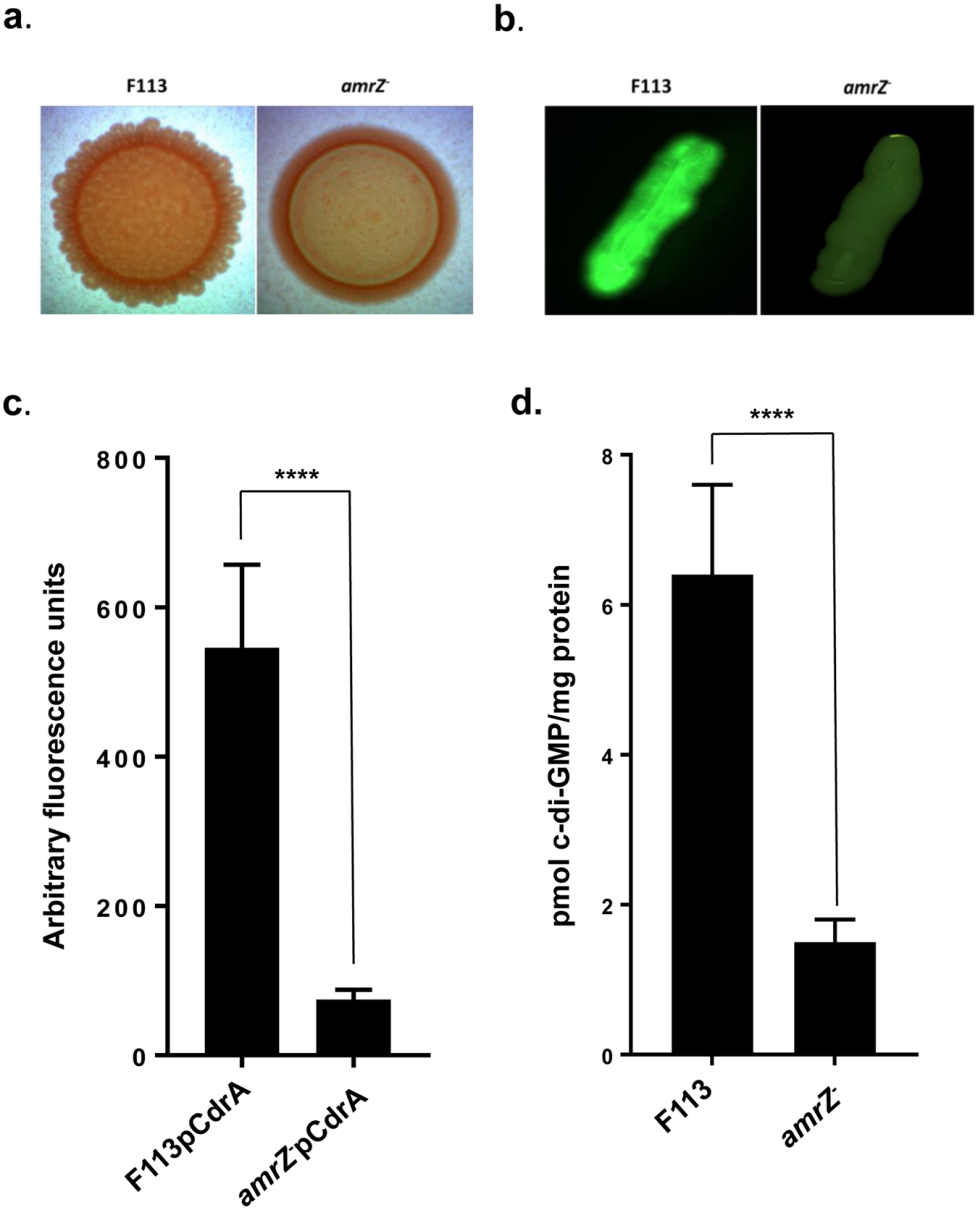
AmrZ regulates exopolysaccharide production and c-di-GMP levels. (**a**) Morphology of colonies of *P. fluorescens* F113 and its *amrZ* mutant after 48 h growth in King’s B medium in the presence of Congo Red. (**b**) Streaks on LB medium of *P. fluorescens* F113 and its *amrZ* mutant harboring a plasmid with the *gfp* based pCdrA biosensor for c-di-GMP. Pictures were taken with a Leika binocular microscope, using a GFP filter set. Exposition time was 50 milliseconds. (**c**) Quantification of fluorescence emitted by *P. fluorescens* F113pCdrA and its *amrZ* mutant harboring pCdrA. Fluorescence of the *amrZ* mutant harboring pCdrA was referred to the fluorescence of the F113pCdrA strain. Averages and standard deviations of three independent experiments with sixteen technical replicas each are presented. (**d**) c-di-GMP quantification by LC-MS in *P. fluorescens* F113 and its *amrZ* mutant. Averages and standard deviation of five analyzed extracts per strain are presented. Asteriks represent statistical significance of the data: ****p < 0.0001.

Motility and biofilm formation are inversely regulated by the messenger molecule c-di-GMP. This molecule also regulates exopolysaccharides production. To test whether AmrZ affects exopolysaccharide production, the *amrZ* mutant and the wt strain were grown in King’s B medium in the presence of Congo Red, a dye that binds to several exopolysaccharides and has been used as an indirect measurement of c-di-GMP production. As shown in Fig. 2a, the two strains showed a different colouring pattern and colony morphology in the presence of Congo Red, indicating different exopolysaccharide(s) production. To test the cytoplasmic concentration of c-di-GMP, two methods were used. First, introduction of the *gfp* based pCdrA biosensor^19^ in the wt strain and the *amrZ* mutant revealed that c-di-GMP concentration was higher in the wt strain (Fig. 2b). Quantification of fluorescence (Fig. 2c) confirmed that the wild-type strain contained about five times more c-di-GMP than the *amrZ* mutant. These results were validated by determination of c-di-GMP concentration in both strains through LC-MS (Fig. 2d), showing that c-di-GMP concentration in the wt was 6.4 pmol/mg protein compared to the 1.5 pmol/mg protein measured in the *amrZ* mutant. These results show that c-di-GMP concentration is 4.2 times higher in the wt, indicating that AmrZ is a major determinant of c-di-GMP levels in *P. fluorescens* F113.

### AmrZ transcriptionally regulates a large number of genes implicated in c-di-GMP turnover

**Figure 3:**
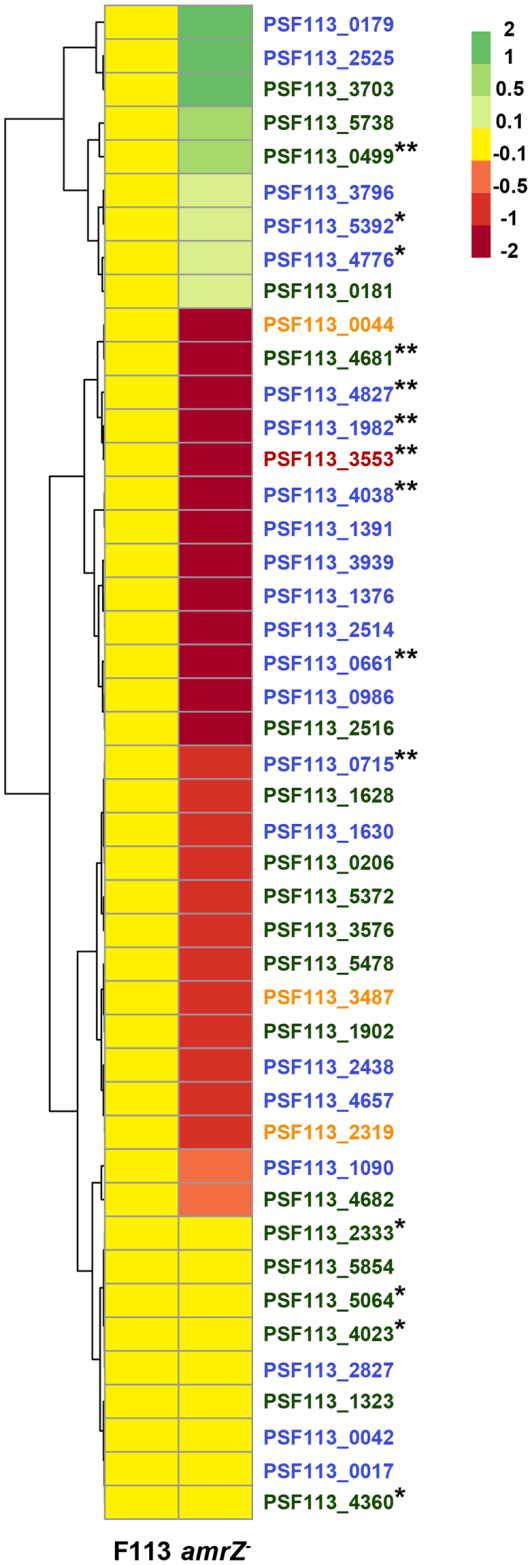
AmrZ transcriptionally regulates a large number of genes implicated in c-di-GMP turnover. Heatmap showing the differential expression of all the *P. fluorescens* F113 genes encoding enzymes involved in c-di-GMP turnover, in the wild-type strain and the *amrZ* mutant, in stationary phase. The heatmap was constructed based on FPKM values obtained by RNA-Seq data. The four different colors of the gene legends, depend on the protein domains: blue, GGDEF; orange, EAL; green, GGDEF and EAL; red, HD-GYP. The color scale represents the log base 2 of fold change of each gene relative to the wild-type: green, genes repressed by AmrZ, red-orange, genes upregulated by AmrZ; yellow, no AmrZ regulation. Log_2_ fold change values and p and q values for each gene are provided in Supplementary Table 1. *AmrZ binding site in gene promoter; **AmrZ binding site in gene promoter and expression modified in *amrZ* mutant (p-value < 0.003).

We have previously shown that AmrZ binds to the promoter region of 14 genes implicated in c-di-GMP turnover^1^ (Table 1), which are therefore strong candidates to be directly regulated by AmrZ. We used an RNA-Seq approach to determine the influence of AmrZ in the expression of all the c-di-GMP turn-over related genes encoded in the F113 genome. Expression data showed the effects of AmrZ on the regulation of c-di-GMP genes. This regulation was especially evident during stationary phase, as shown in Fig. 3. During this phase, the expression of most of the c-di-GMP genes is affected by the lack of the *amrZ* gene. Out of the affected genes, five were shown to be repressed by AmrZ, since they showed higher expression in the *amrZ* mutant than in the wild-type strain. Conversely, twenty five c-di-GMP related genes were shown to be positively regulated by AmrZ, since their expression was lower in the *amrZ* mutant than in the wild-type. These results show that the expression of more than 65% of the c-di-GMP genes encoded in the F113 genome is affected by AmrZ, which acts mostly as a transcriptional activator for these genes. Furthermore, eight of the genes that were predicted as putative direct targets of AmrZ^1^, were indeed shown to be regulated by this protein (p < 0.003), seven of them positively and one of them negatively. Complete results for all the c-di-GMP genes (fold activation/repression, p and q values) are shown in Supplementary Table 1.

**Table 1:** c-di-GMP related genes identified as putative AmrZ binding targets.

Locus	Name or homologue	c-di-GMP relates domains	Reference
PSF113_0499	*dipA* (PA5017)	GGDEF & EAL	^30^
PSF113_0661	*gcbA* (Pfl01_0623)	GGDEF	^29^
PSF113_0715	*yfiN* (PA1120)	GGDEF	^48^
PSF113_1982	*adrA* (PFL_4532)	GGDEF	^49^
PSF113_2333	*mucR* (PA1727)	GGDEF	^50^
PSF113_3553	PA4108	HD-GYP	—
PSF113_4023	PFL_1902	GGDEF & EAL	^49^
PSF113_4038	*gcbB* (Pfl01_1790)	GGDEF	^29^
PSF113_4360	Pfl01_1678	GGDEF & EAL	^29^
PSF113_4681	Pfl01_4551	GGDEF & EAL	^29^
PSF113_4776	*gcbC* (Pfl01_4666)	GGDEF	^29^
PSF113_4827	—	GGDEF	—
PSF113_5064	*morA* (PA4601)	GGDEF & EAL	^51^
PSF113_5392	PFL_5686	GGDEF	^49^

### Most of the c-di-GMP related genes directly regulated by AmrZ are involved in motility and/or biofilm formation

**Figure 4:**
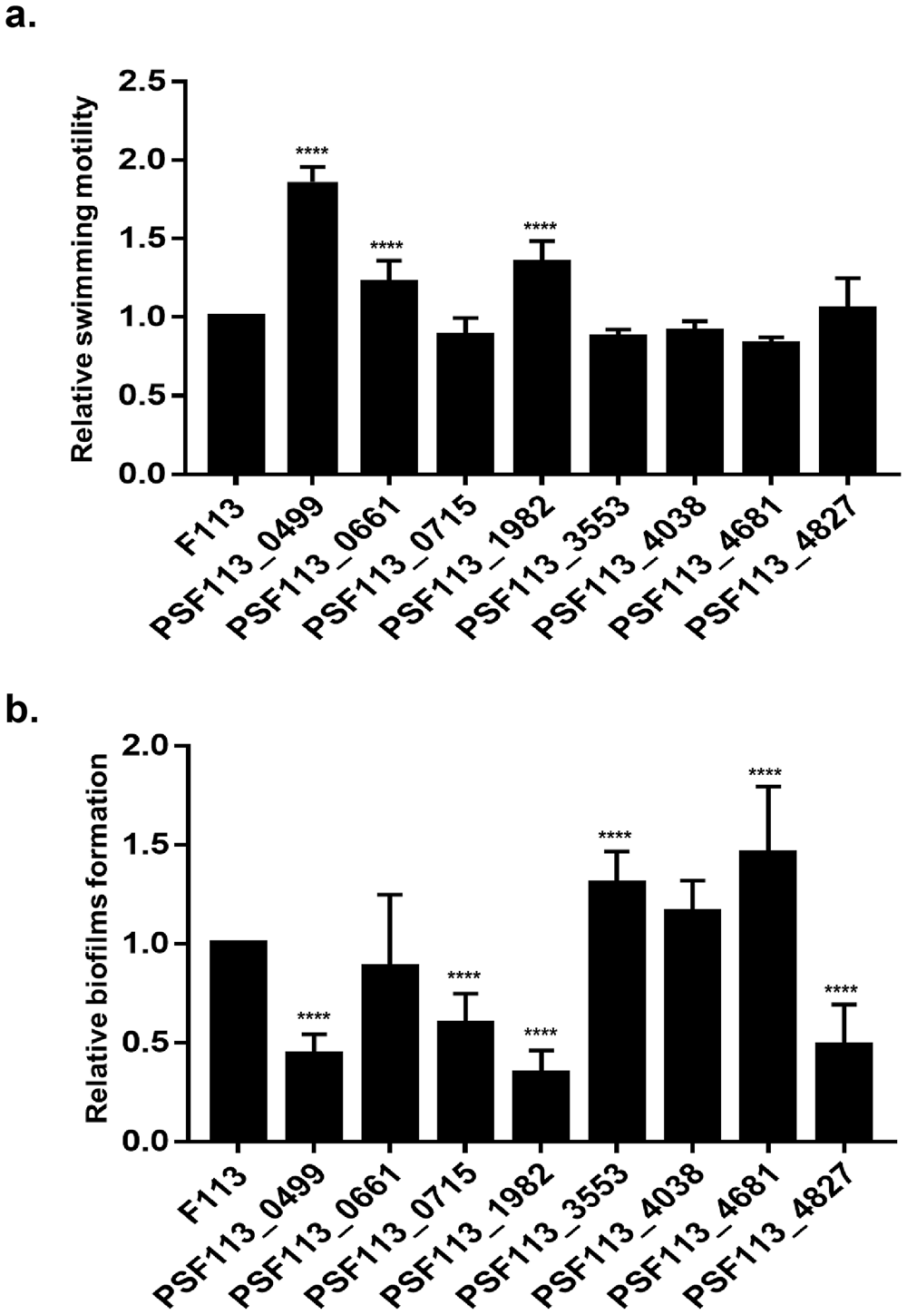
Most of the c-di-GMP related genes that are directly regulated by AmrZ are involved in motility and/or biofilm formation. (**a**) Relative swimming motility phenotype of *P. fluorescens* F113 and its eight mutants affected in the indicated gene (ORF number in www.pseudomonas.com)^47^. Experiments and replicas were performed as in Fig. 1. (**b**) Relative biofilm formation (attachment) phenotype of the same strains above. Experiments and replicas were performed as in Fig. 1. Asterisks represent statistical significance of the data: ****p < 0.0001.

In order to determine roles for the AmrZ-regulated c-di-GMP genes in motility and biofilm formation, we constructed mutants in the eight c-di-GMP genes that were predicted to be AmrZ binding-targets by ChIP-Seq^1^ and also showed regulation by AmrZ by RNA-Seq. All the mutants were tested for swimming motility (Fig. 4a) and biofilm formation (Fig. 4b). Seven out of the eight mutants showed a motility and/or biofilm formation phenotype compared to wt F113. The mutant affected in the PsF113_ 4038 gene (*gcbB*), did not show any significant phenotype. Only three of the mutants, those affected in genes PsF113_0499 (*dipA*), PsF113_0661 (*gcbA*) and PsF113_1982 (*adrA*) had a swimming motility phenotype, all of them showing increased motility. Six of the mutants had a significant biofilm formation phenotype: two of them increased biofilm formation and four of them showed a significant reduction in biofilm formation. Two of the mutants affected in motility, *dipA*^*−*^ and *adrA*^*−*^ were also affected in biofilm formation, showing increased motility and reduced biofilm formation phenotypes. The swimming motility phenotypes of *gcbA* and *adrA* mutants and the biofilm formation phenotype of the *adrA* mutant were complemented by expressing a wild-type copy of the respective gene under a constitutive promoter on a plasmid (Supplementary Fig. 2). However, we were unable to complement any of the phenotypes of the *dipA* mutant.

## Discussion

AmrZ has been shown to be a global regulator in *Pseudomonas fluorescens*^1^ and *P. aeruginosa*^6^. This global role leads to pleiotropic phenotypes of mutants lacking this regulator. As shown in Fig. 1, the F113 *amrZ* mutant is affected in motility and biofilm formation, being hypermotile and defective in biofilm formation. Although the hypermotility phenotype is also present in the *P. aeruginosa* mutant, the biofilm formation phenotypes are opposed: defective in *P. fluorescens* and enhanced in *P. aeruginosa*. The hyperbiofilm formation in the *P. aeruginosa* mutant was originally attributed to the overproduction of the *psl* exopolysaccharide^20^, not produced by F113, but was later related also to c-di-GMP levels^6^. An interesting phenotype of the F113 *amrZ* mutant is its compromised ability to competitively colonize the rhizosphere. F113 hypermotile mutants described earlier were hypercompetitive for rhizosphere colonization^21^, independently of their ability to form biofilms^18^. In turn, the rhizosphere colonization phenotype of the hypermotile *amrZ* mutant is similar to those mutants that have lost motility^22^ or chemotaxis^23^. These results indicate that factors besides motility and biofilm formation are affected in the *amrZ* mutant and that these factors are important for the fitness of *P. fluorescens* in the rhizosphere environment. It is interesting to note that the *P. aeruginosa amrZ* mutant is less virulent than the wild-type strain in a murine system and that this lack of virulence is associated to reduced-lung colonization ability after intranasal inoculation^24^. Considering the overlap of the AmrZ regulon in both strains^1,6^ it is likely that AmrZ plays a similar role in niche adaption in the rhizosphere and the lung.

Attachment and biofilm formation defects in pseudomonads are associated to defects in exopolysaccharides production and low levels of c-di-GMP. The F113 *amrZ* mutant forms reduced amounts of biofilm when grown in glass tubes and shows reduced attachment to plastic in microtiter plate assays. In order to test for differences in exopolysaccharides production, a Congo Red binding-assay was used. Congo Red binds to different extracellular components including several polysaccharides such as cellulose and to amyloid proteins^25,26^. The results presented here, show a different colony morphology and coloring pattern for the wild-type strain and the *amrZ* mutant, indicating alterations in cellular envelopes. However, since the F113 genome does not encode genes for cellulose, *pel* or *psl* synthesis, the exopolysaccharides produced by *P. aeruginosa* and implicated in biofilm formation, the differences observed in F113 should be due to differences in the production of other polysaccharides or other extracellular components. Regarding c-di-GMP levels, the results presented here show that the F113 mutant has severely reduced levels of cytoplasmic c-di-GMP, as revealed by two independent methods: a fluorescent biosensor and direct measurements by LC-MS. These findings fit well with the paradigmatic role of c-di-GMP in determining bacterial life-style, since low levels of c-di-GMP are related to high motility and reduced biofilm formation, supporting a planktonic rather than sessile life-style^9^. However, the phenotypes of c-di-GMP levels in the F113 *amrZ* mutant and the *P. aeruginosa* mutant are opposed, since the *P. aeruginosa amrZ* mutant contains more than two fold more c-di-GMP than the wild-type^6^. In the case of *P. aeruginosa*, these c-di-GMP levels are in concordance with the hyperbiofilm formation phenotype of the *amrZ* mutant. On the contrary, the high levels of c-di-GMP do not explain the hypermotility phenotype of the mutant. This apparent contradiction might be explained by the direct transcriptional repression of motility genes by AmrZ, observed both in *P. aeruginosa*^6^ and *P. fluorescens* F113^27,28^.

AmrZ is therefore a major determinant of c-di-GMP levels in *P. fluorescens* and *P. aeruginosa*. We have shown here that in F113, AmrZ influences the transcription of a majority of the genes encoding enzymes implicated in c-di-GMP turnover. Regulation of c-di-GMP related genes by AmrZ in this strain is mostly transcriptional activation, although five genes, two of them encoding GGDEF proteins and the other three GGDEF-EAL proteins, seem to be repressed by AmrZ. Among the twenty five genes transcriptionally activated by AmrZ, thirteen encode GGDEF, eight GGDEF-EAL, three EAL and one HD-GYP proteins. These results show that in F113, c-di-GMP levels are controlled by AmrZ in a complex regulatory network and that the low levels of c-di-GMP observed in the *amrZ* mutant are the result of a balance between activation and repression of a large number of genes encoding DGCs and PDEs. These findings contrast with those in *P. aeruginosa* where most of the high c-di-GMP level was attributed to the repression of a single gene encoding a DGC^6^. Regarding the fourteen c-di-GMP related genes previously identified as putative targets of AmrZ by ChIP-Seq^1^, RNA-Seq has confirmed the regulation by AmrZ of eight of them. Six of them showed expression levels that were more than two fold higher than in the wild-type strain indicating that activation was the general trend. Conversely, only one (*dipA*) was shown to be repressed by AmrZ. For three of the genes identified by ChIP-Seq but not appearing as regulated in the RNA-Seq experiment, very low expression, around the limit of detection was observed under the experimental conditions tested.

Phenotypic analysis of the eight mutants affected in genes confirmed to be directly regulated by AmrZ, has implicated most of them in biofilm formation, while only three were affected in motility. Two out of these three were affected both in motility and biofilm formation. These results are very similar to those observed in a systematic analysis of the phenotypes of 30 putative DGCs encoded in the *P. fluorescens* Pf-01 genome^29^, indicating that different c-di-GMP pools, generated and degraded by different enzymes, impact on different pathways for motility and biofilm formation. It is interesting to note that one of these genes did not show any significant role in motility or biofilm formation, at least under our experimental conditions, suggesting that it might be implicated in the regulation of other traits.

We have also found a correlation between motility and biofilm phenotypes and the domains present in the encoded proteins: All of the predicted DGCs (increasing motility and/or decreasing biofilm formation in the mutants), contained a GGDEF motif. Similar results were found with respect to PDEs, since all contained an EAL or HD-GYP motif. On the other hand, phenotypic typing of PsF113_0499, indicates that this gene encodes a DGC. However, this gene is an orthologue of *dipA*, a gene that has been shown to encode a PDE in *P. aeruginosa*^30^ and possesses a highly degenerated GGDEF motif, unlikely to be catalytic. It is important to remark that we were unable to complement either the biofilm or the motility phenotypes of this mutant with a wild-type copy of the gene, indicating an indirect or gene-copy dependent regulation of these traits by this gene. In *P. aeruginosa* two c-di-GMP related genes have also been shown as targets of AmrZ^6^. One of these genes, encoding the DGC AdcA (also named GcbA) is an orthologue of the AmrZ regulated PsF113_0661. However, while this gene is AmrZ-repressed in *P. aeruginosa*, it is AmrZ-activated in F113. It is interesting to note that not only genes encoding c-di-GMP enzymes, but also genes encoding sensors or receptors for this molecule such as the PilZ protein FlgZ^28^ and its *P. aeruginosa* orthologue are also predicted targets of AmrZ, both in *P. fluorescens*^1^ and in *P. aeruginosa*^6^, suggesting that c-di-GMP sensing is also implicated in AmrZ regulation.

## Conclusions

AmrZ is a global regulator required for niche adaption in *Pseudomonas fluorescens* and *P. aeruginosa*. An important part of AmrZ activity is to modulate the intracellular levels of c-di-GMP, which in turn regulates swimming motility and biofilm formation, among other adaptive traits. While in *P. aeruginosa* AmrZ contributes to a lowering in c-di-GMP levels, in *P. fluorescens* AmrZ increases the levels of the messenger molecule. Taken together, the results presented here show that although AmrZ is a major determinant of c-di-GMP levels by regulating the expression of genes encoding DGCs and PDEs in *P. fluorescens* F113 and *P. aeruginosa* PAO1, regulation is very different in both strains. While in PAO1 only one gene encoding a DGC and one encoding a PDE are transcriptionally repressed, in F113 c-di-GMP levels are the consequence of activation and repression of a complex network of genes encoding many of the DGCs and PDEs present in the bacterium.

## Materials and Methods

### Bacterial strains, plasmids and mutant construction

*Pseudomonas fluorescens* F113 and derivatives were routinely grown in SA^31^ medium at 28 °C. Purified agar (1.5%) was used for solid medium and antibiotics were used for selection as required (rifampicin (Rif), 100 µg/mL; tetracycline (Tet), 70 µg/mL; kanamycin (Km), 50 µg/mL; cicloheximide (Clh), 10 µg/mL, gentamicin (Gm), 3 µg/mL and piperacillin (Pip) 30 µg/mL). To induce expression of vector promoters, IPTG 0.25 mM was used. *E. coli* DH5α was used for DNA manipulation and was grown at 37 °C in LB medium supplemented with bacteriological agar (1.5%) and/or antibiotics when required (ampicillin (Amp), 100 µg/mL; tetracycline (Tet), 10 µg/mL; kanamycin (Km), 25 µg/mL; gentamicin (Gm), 10 µg/mL; piperacillin (Pip) 30 µg/mL and chloramphenicol 30 µg/mL).

Mutants were constructed by homologous recombination of internal fragments of the targeted gene, cloned into the suicide vectors pK18*mobsacB*^32^ or pG18*mob2*^33^. The constructs were introduced into F113 by triparental mating and selected for homologous recombination. Mutant construction is detailed in Supplementary Table 1. All mutants were checked by PCR and Southern blotting. Complementation of mutants was performed by expressing a wild-type copy of the gene in expression vectors pVLT31^34^ or pBBRMCS5^35^ (Supplementary Table 1).

### Phenotypic analysis of mutants

Swimming motility was determined in SA plates supplemented with 0.3% of purified agar as described before^36^. Swimming halo diameters were determined after 24 h incubation at 28 °C. Experiments were performed in triplicate, with three replicas in each experiment.

To examine biofilm formation a modified version of a previously described quantification method of Peeters *et al*. was used^37^. Briefly, overnight cultures grown in LB medium were adjusted to OD_600_ at 0.8 into a fresh LB and incubated at 28 °C for 2 h in 96-well-microlitre plates. After staining and washing, absorbance of the eluted crystal violet was measured at OD_590_ on a Synergy HT multi-mode microplate reader (BioTek, Wilusky, VT, USA). Experiments were repeated three times with 16 technical replicas in each assay.

Competitive rhizosphere colonization analysis was performed essentially as described in^38^ using alfalfa (*Medicago sativa* var. Resis) seedlings. *P. fluorescens* Km^r^ was used as a control^22^. Control and tester strains were co-inoculated at 1 × 10^3^ colony-forming units (CFUs) to one-week-old seedlings. After a further week, rhizosphere bacteria were recovered by vortexing and dilutions plated on SA agar with Rif and Clh. CFU of each strain were distinguished and counted by growth with and without antibiotic. Experiments were performed in triplicate and each experiment contained eight independent plants.

Congo Red binding and colony morphology was determined on King’s B plates^39^ supplemented with 0.004% Congo Red. 10 µL drops of an overnight culture were spotted on plates and incubated at 28 °C for 48 h before inspection.

### c-di-GMP determinations

Fluorescence intensity emitted by F113 and *amrZ*^*−*^ strains harbouring the pCdrA::*gfp*^C^ biosensor vector^19^ was visualized with a Leica M165 FC stereomicroscope using a GFP filter set (Excitation/Emission 494/518 nm) with different exposure times. Pictures were taken with the Leica Application Suite software, with exposure time elapsed to 50 milliseconds.

Indirect quantitative detection of c-di-GMP intracellular concentration was measured by microplate-based assays of the wt and *amrZ*^*−*^ strains harbouring pCdrA::*gfp*^C^ vector. Overnight cultures grown in LB were diluted to OD_600_ at 0.5 and fluorescence (excited at 485/20, emission at 528/20 nm) was measured in a Synergy HT multi-mode microplate reader (BioTek, Wilusky, VT, USA) using 96 black-well plates. The experiments were conducted in triplicate with 16 technical replicas in each experiment.

Extraction and quantification of c-di-GMP was performed as described in^40^. Extraction was performed from 50 mL of LB cultures grown to OD_600_ of 0.6. Pellets were used for protein determination by A_280_ in a NanoDrop spectrophotometer, after solubilization in 200 µL of 0.1 M NaOH and heating at 95 °C for 15 min. Nucleotide extracts were subjected to LC-MS as described^40^ and c-di-GMP was quantified by using an external standard calibration curve. Five extracts were analyzed for each strain.

### Statistical analysis

For all the tested phenotypes, results were statistically analyzed using GraphPad Prism version 7.00 (GraphPad software, La Jolla, California, USA). Data were compared using one way analysis of variance (ANOVA) followed by Tukey´s correction of multiple comparison test (p ≤ 0.0001) or unpaired t test two-tailed (p ≤ 0.0001).

### RNA extraction and RNA-Seq analysis

RNA was extracted from cultures grown in SA medium to exponential (OD_600_ 0.6) and stationary phase (OD_600_ 1.2). Three independent cultures per strain and condition were mixed prior to RNA extraction. RNA isolation was performed using TRIzol^TM^ (Invitrogen)-chloroform followed by an isopropanol precipitation. The obtained RNA was treated with DNase (RQ1, Promega) and finally was purified using the RNAclean and Concentrator^TM^-5 column (Zymo Research). rRNA depletion, library construction and sequencing was custom performed by Sistemas Genómicos (Paterna, Valencia). Two libraries per sample were prepared and sequencing was carried out using Illumina MiSeq paired end, 2 × 100 bp.

Illumina reads were clipped and trimmed to remove low quality nucleotides as well as putative Illumina adapters by using Trimmomatic v 0.35^41^, specifying a sliding window of 4 nts with an average phred quality of 20 and 50 nts as minimum read length to be conserved. Filtered paired reads were aligned to reference genome *P. fluorescens* F113 (GenBank: NC_016830) with Bowtie v2^42^. Alignment SAM files were sorted and converted to BAM using SAMtools^43^ and gene counts were determined by HTSeq^44^. HTSeq-count tables were used to calculate expression levels for each gene using FPKM formula^45^ and statistical significance using DESeq 2 R package^46^. Relative expression values were calculated as follows log_2_ (FPKM^AmrZ^) − log_2_(FPKM^F113^). *P. fluorescens* F113 genes annotated as encoding diguanylate cyclases and phosphodiesterases were selected to prepare the heatmap expression figure with pheatmap R package.

### Data availability

RNA-Seq raw data have been deposited at NCBI as Bioproject PRJNA419480.

## Electronic supplementary material

Supplementary Information
