# Supplementary material for: AmrZ is a major determinant of c-di-GMP levels in Pseudomonas fluorescens F113
Source: Sci Rep. 2018 Jan 31;8:1979. doi: 10.1038/s41598-018-20419-9 (PMC5792552; doi:10.1038/s41598-018-20419-9)
Supplement: Supplementary file 1 — Supplementary Information [file 41598_2018_20419_MOESM1_ESM.pdf]

SUPPLEMENTARY INFORMATION

AmrZ is a major determinant of c-di-GMP levels in *Pseudomonas fluorescens* F113

Candela Muriel[1], Eva Arrebola[1], Miguel Redondo-Nieto[1], Francisco Martínez-Granero[1], Blanca Jalvo[1], Sebastian Pfeilmeier[2], Esther Blanco-Romero[1], Irene Baena[1], Jacob G. Malone[2], Rafael Rivilla[1] and Marta Martín[1]*

1. Departamento de Biología. Universidad Autónoma de Madrid. Darwin, 2. 28034 Madrid. Spain.
2. Department of Molecular Microbiology. John Innes Centre. Colney Lane, NR47UH. Norwich. UK

Supplementary Table S1: Strains and Plasmids

Strains	Description	Reference
P. fluorescens		
F113	*P. fluorescens* F113 wild type, Rif^R	(1)
amrZ$^-$	F113*amrZ*$^-$, Rif^R Km^R	(2)
PSF113_0499	F1130049$^-$, Rif^R Km^R	This work
PSF113_0661	F1130661$^-$, Rif^R Km^R	This work
PSF113_0715	F1130715$^-$, Rif^R Km^R	This work
PSF113_1982	F1131982$^-$, Rif^R Km^R	This work
PSF113_3553	F1133553$^-$, Rif^R Km^R	This work
PSF113_4038	F1134038$^-$, Rif^R Km^R	This work
PSF113_4681	F1134681$^-$, Rif^R Km^R	This work
PSF113_4827	F1134827$^-$, Rif^R Km^R	This work
E. coli		
DH5α	*E. coli* cloning strain*: Φ80lacZΔM15, Δ(lacZYA-argF), U169, recA1, endA1, hsdR17 (rK,mK+), phoA, supE44-λ–thi-1,gyrA96, relA1*	Gibco-BRL
**Plasmids**		
pCR2.1 TOPO	Cloning vector, Km^R	Life Technologies
pGEM T-easy	Cloning vector, Amp^R	Promega Corp.
pK18*mobsacB*	Suicide vector for allelic exchange; pUC18 derivative *lacZ mob* site *sacB*, Km^R	(3)
pG18 *mob2*	Suicide vector *sacB,* Gn^R	(4)
pVLT31	IPTG-inducible expression vector, Tet^R	(5)
pBBRMCS-5	Replicative plasmid with *lacZ* promoter for overexpression, Gn^R	(6)
pRK600	Helper plasmid Cm^R	(7)
pBG1759	pVLT31 containing the *amrZ* gene (p*amrZ*), Tet^R	This work
pBG2059	pK18*mobsacB* derivative with a 500 bp SalI-SphI fragment of F1130499 gene, Km^R	This work
pBG2081	pK18*mobsacB* derivative with a 690 bp EcoRI fragment of F1130661 gene, Km^R	This work
pBG2092	pK18*mobsacB* derivative with a 500 bp EcoRI fragment of F1130715 gene, Km^R	This work
pBG2101	pK18*mobsacB* derivative with a 518 bp EcoRI fragment of F1131982 gene, Km^R	This work
pBG2099	pK18*mobsacB* derivative with a 675 bp EcoRI fragment of F1133553 gene, Km^R	This work
pBG2086	pK18*mobsacB* derivative with a 672 bp EcoRI fragment of F1134038 gene, Km^R	This work
pBG2090	pK18*mobsacB* derivative with a 1200 bp EcoRI fragment of F1134681 gene, Km^R	This work
pBG2027	pG18*mob2* derivative with a 700 bp EcoRI fragment of F1134827 gene, Km^R	This work
pBG2065	pBBRMCS-5 with 0499 gene cloned at HindIII/XbaI site, Gn^R	This work
pBG2135	pBBRMCS-5 with 0661 gene cloned at HindIII/XbaI site, Gn^R	This work
pBG2128	pBBRMCS-5 with 1982 gene cloned at KpnI/HindIII site, Gn^R	This work

References

1. Shanahan, P., O'Sullivan D, J., Simpson, P., Glennon, J. D. & O'Gara, F. Isolation of 2,4-diacetylphloroglucinol from a fluorescent pseudomonad and investigation of physiological parameters influencing its production. *Appl Environ Microbiol* **58**, 353-358 (1992).

2. Martinez-Granero, F. *et al.* The Gac-Rsm and SadB signal transduction pathways converge on AlgU to downregulate motility in *Pseudomonas fluorescens*. *PLoS One* **7**, e31765, doi:10.1371/journal.pone.0031765 (2012).

3. Schäfer, A. et al. Small mobilizable multi-purpose cloning vectors derived from the *Escherichia coli* plasmids pK18 and pK19: selection of defined deletions in the chromosome of *Corynebacterium glutamicum*. *Gene* **145**, 69-73 (1994).

4. Kirchner, O. & Tauch, A. Tools for genetic engineering in the amino acid-producing bacterium *Corynebacterium glutamicum*. *Journal of biotechnology* **104**, 287-299 (2003).

5. de Lorenzo, V., Eltis, L., Kessler, B. & Timmis, K. N. Analysis of Pseudomonas gene products using lacIq/Ptrp-lac plasmids and transposons that confer conditional phenotypes. *Gene* **123**, 17-24 (1993).

6. Kovach, M. E. *et al.* Four new derivatives of the broad-host-range cloning vector pBBR1MCS, carrying different antibiotic-resistance cassettes. *Gene* **166**, 175-176 (1995).

7. Finan, T. M., Kunkel, B., De Vos, G. F. & Signer, E. R. Second symbiotic megaplasmid in *Rhizobium meliloti* carrying exopolysaccharide and thiamine synthesis genes. *Journal of bacteriology* **167**, 66-72 (1986).

Supplementary Table 2. AmrZ transcriptional regulation of genes implicated in c-di-GMP turnover. Log2 Fold change of *amrZ* mutant expression against F113 wild-type in all genes with diguanylate cyclases and/or phosphodiesterase domains. Statistical significance is shown by p and q values. N.A.: Not Available

Domain	Locus	log2FC	p-value	q-value
EAL	PSF113_0044	-1.852801854	4.514E-51	5.10767E-50
EAL	PSF113_3487	-0.906837059	3.42484E-10	1.33594E-09
EAL	PSF113_2319	-0.721411243	1.10679E-08	3.94189E-08
GGDEF + EAL	PSF113_4681	-1.793986383	0	0
GGDEF + EAL	PSF113_2516	-1.370520403	7.1204E-235	3.5395E-233
GGDEF + EAL	PSF113_5478	-0.89854943	2.7552E-96	5.39536E-95
GGDEF + EAL	PSF113_1902	-0.831517375	7.36236E-45	7.45984E-44
GGDEF + EAL	PSF113_3576	-0.684428407	2.87732E-54	3.4152E-53
GGDEF + EAL	PSF113_5372	-0.681158131	6.761E-49	7.36366E-48
GGDEF + EAL	PSF113_0206	-0.66618906	4.81523E-10	1.86454E-09
GGDEF + EAL	PSF113_1628	-0.629662824	1.9859E-65	2.79661E-64
GGDEF + EAL	PSF113_4682	-0.390844676	2.07592E-25	1.38142E-24
GGDEF + EAL	PSF113_5064	-0.047522371	0.518449564	0.627926419
GGDEF + EAL	PSF113_5854	-0.047262242	0.700614696	0.781956452
GGDEF + EAL	PSF113_1323	-0.025626022	0.636232474	0.731001199
GGDEF + EAL	PSF113_4023	0.105646332	0.106127516	0.169291727
GGDEF + EAL	PSF113_4360	0.107343568	0.68059559	0.766823702
GGDEF + EAL	PSF113_2333	0.110695086	0.444282901	0.560874615
GGDEF + EAL	PSF113_0181	0.142150889	0.118124501	0.185930202
GGDEF + EAL	PSF113_0499	0.538604403	6.90144E-16	3.41316E-15
GGDEF + EAL	PSF113_5738	0.747956746	6.70964E-10	2.5809E-09
GGDEF + EAL	PSF113_3703	1.43989134	2.6272E-32	2.04549E-31
GGGDEF	PSF113_4827	-1.698102576	8.354E-137	2.2704E-135
GGGDEF	PSF113_0661	-1.52776552	1.872E-89	3.42375E-88
GGGDEF	PSF113_1982	-1.515026842	4.41271E-54	5.20575E-53
GGGDEF	PSF113_1376	-1.44544019	3.7312E-75	5.89693E-74
GGGDEF	PSF113_2514	-1.388611892	5.13796E-72	7.88454E-71
GGGDEF	PSF113_0986	-1.189448679	0.262071851	N.A.
GGGDEF	PSF113_4038	-1.171864704	2.7733E-39	2.49683E-38
GGGDEF	PSF113_1391	-1.066982072	1.37202E-11	5.74077E-11
GGGDEF	PSF113_3939	-1.041108731	8.7103E-19	4.76567E-18
GGGDEF	PSF113_2438	-0.784777602	8.24607E-23	5.10204E-22
GGGDEF	PSF113_4657	-0.680393512	7.19976E-08	2.44162E-07
GGGDEF	PSF113_1630	-0.667439514	6.87376E-72	1.05205E-70
GGGDEF	PSF113_2827	-0.489219108	0.585035853	N.A.
GGGDEF	PSF113_0715	-0.486246402	0.003245028	0.007031701
GGGDEF	PSF113_1090	-0.17408767	0.080885229	0.133303625
GGGDEF	PSF113_0017	-0.148219873	0.186396758	0.274312637
GGGDEF	PSF113_0042	-0.082923382	0.432304489	0.548610715
GGGDEF	PSF113_4776	0.251289838	0.001757575	0.003952844
GGGDEF	PSF113_3796	0.26475815	0.006274119	0.012948998
GGGDEF	PSF113_5392	0.397196264	0.031163349	0.056480535
GGGDEF	PSF113_0179	1.266635792	1.8496E-110	3.999E-109
GGGDEF	PSF113_2525	1.430122104	1.4973E-42	1.44656E-41
HD-GYP , HDOD	PSF113_3553	-1.601093608	2.82826E-23	1.77063E-22

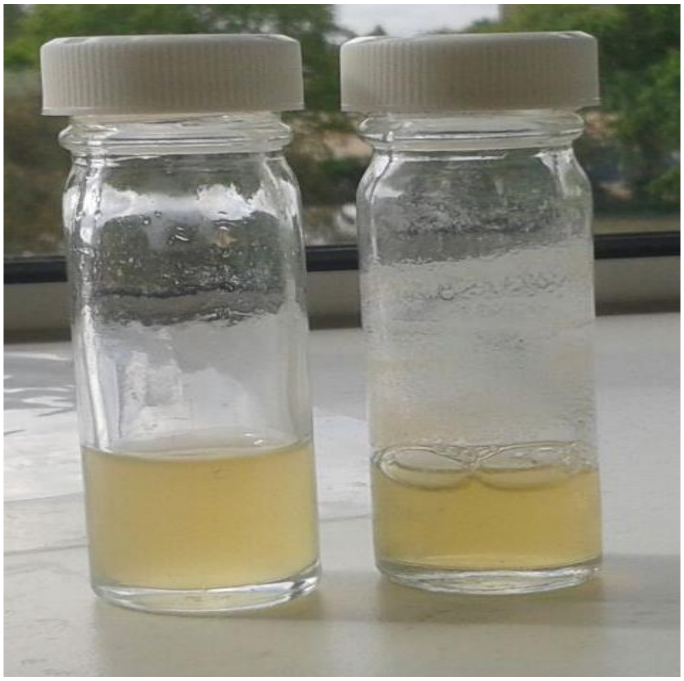

amrZ^{-} F113

Supplementary Fig.1. Biofilm formation on glass tubes

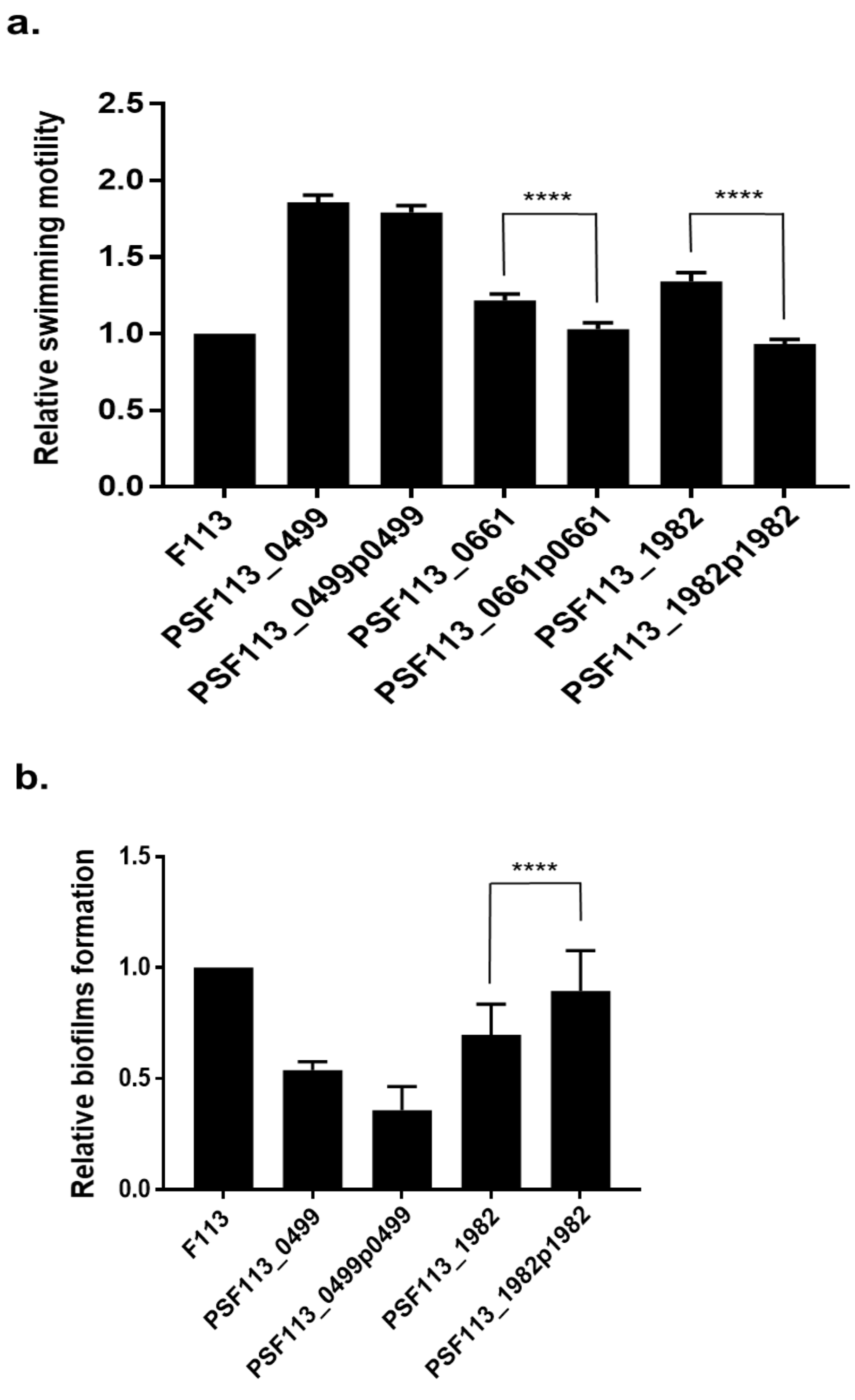

Supplementary figure 2:

Phenotype complementation analysis of the mutants. a. Swimming motility phenotypes of *P. fluorescens* F113, PSF113_0499, PSF113_0661 and PSF113_1982 mutants and complementation analysis of each mutant. b. Biofilm formation (attachment) phenotypes of *P. fluorescens* F113, PSF113_0499 and PSF113_1982 mutants and complementation analysis of each mutant. Experiments and replicas were performed as in Fig. 1. Asterisks represent statistical significance of the data: **** $p<0.0001$.
